# Adrenergic Alpha-1 Pathway Is Associated with Hypertension among Nigerians in a Pathway-focused Analysis

**DOI:** 10.1371/journal.pone.0037145

**Published:** 2012-05-16

**Authors:** Nicholas P. Reder, Bamidele O. Tayo, Babatunde Salako, Adesola Ogunniyi, Adebowale Adeyemo, Charles Rotimi, Richard S. Cooper

**Affiliations:** 1 Department of Preventive Medicine and Epidemiology, Stritch School of Medicine, Loyola University Chicago, Maywood, Illinois, United States of America; 2 Department of Medicine, University of Ibadan, Ibadan, Nigeria; 3 National Institutes of Health Intramural Center for Research on Genomics and Global Health, National Human Genome Research Institute, Bethesda, Maryland, United States of America; Osaka University Graduate School of Medicine, Japan

## Abstract

**Background:**

The pathway-focused association approach offers a hypothesis driven alternative to the agnostic genome-wide association study. Here we apply the pathway-focused approach to an association study of hypertension, systolic blood pressure (SBP), and diastolic blood pressure (DBP) in 1614 Nigerians with genome-wide data.

**Methods and Results:**

Testing of 28 pathways with biological relevance to hypertension, selected *a priori*, containing a total of 101 unique genes and 4,349 unique single-nucleotide polymorphisms (SNPs) showed an association for the adrenergic alpha 1 (ADRA1) receptor pathway with hypertension (p<0.0009) and diastolic blood pressure (p<0.0007). Within the ADRA1 pathway, the genes *PNMT* (hypertension P_gene_<0.004, DBP P_gene_<0.004, and SBP P_gene_<0.009, and *ADRA1B* (hypertension P_gene_<0.005, DBP P_gene_<0.02, and SBP P_gene_<0.02) displayed the strongest associations. Neither *ADRA1B* nor *PNMT* could be the sole mediator of the observed pathway association as the ADRA1 pathway remained significant after removing *ADRA1B*, and other pathways involving *PNMT* did not reach pathway significance.

**Conclusions:**

We conclude that multiple variants in several genes in the ADRA1 pathway led to associations with hypertension and DBP. SNPs in ADRA1B and PNMT have not previously been linked to hypertension in a genome-wide association study, but both genes have shown associations with hypertension through linkage or model organism studies. The identification of moderately significant (10^−2^>p>10^−5^) SNPs offers a novel method for detecting the “missing heritability” of hypertension. These findings warrant further studies in similar and other populations to assess the generalizability of our results, and illustrate the potential of the pathway-focused approach to investigate genetic variation in hypertension.

## Introduction

The high prevalence [1] and heritability [2] of hypertension made it a target for genome-wide association study (GWAS) after the completion of the Human Genome Project, with the hope of developing diagnostic tools or gene-based designer drugs [3]. GWAS have led to the discovery of many single nucleotide polymorphisms (SNPs) associated with various traits and diseases [4]. However, the stiff penalties for testing hundreds of thousands of SNPs may obscure true associations, and GWAS have an increased chance for finding false positive associations if underpowered [5]. The results of hypertension GWAS in particular have been relatively disappointing [6], revealing a small number of SNPs with modest effect sizes. Proposed explanations for the lack of success include the need to identify rare variants with large effect sizes, lack of statistical power to detect common variants with very small effect sizes, error associated with phenotyping, and complex genetic interaction networks.

Due to the relative lack of success of GWAS, alternative methods of identifying the genetic underpinnings of hypertension are being explored, including pathway analysis of GWAS results [7] and targeting of candidate genes [8]. The former approach is a traditional GWAS with the addition of an algorithm that identifies biological pathways with more significant SNPs than would be expected by chance. The latter approach uses genes that have been identified as related to the disease in animal models, Mendelian forms of the disease, or through previous association studies. Both of these approaches have limitations. Pathway analysis of GWAS results can identify pathways that defy biological explanation, while candidate gene studies cannot be used to examine disease-related pathways because they do not include all genes involved in the pathway.

**Figure 1:**
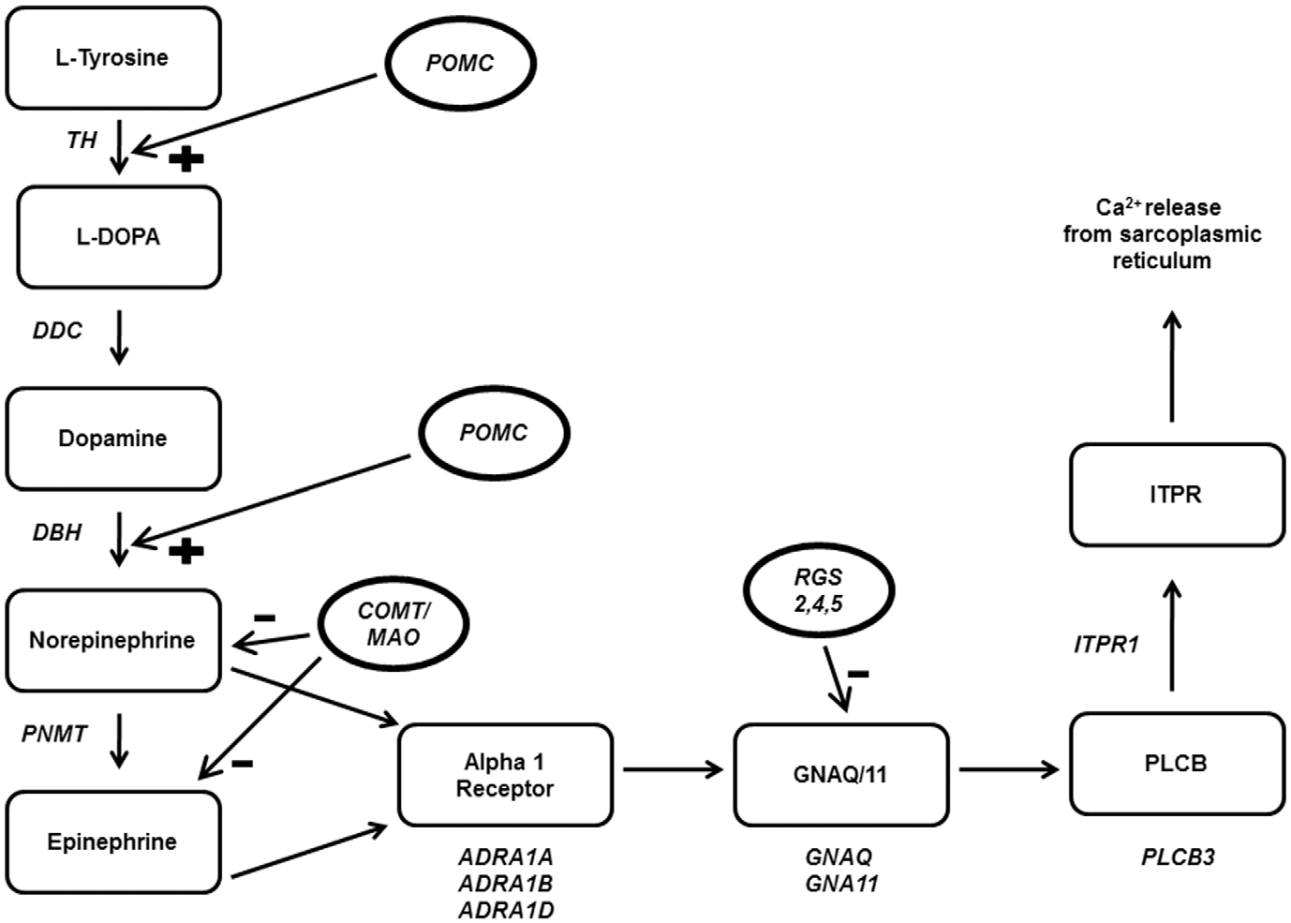
ADRA1 pathway associated genes. Gene names are in italics. Regulators are in circles. TH, tyrosine hydroxylase; DDC, dopa decarboxylase; DBH, dopamine beta hydroxylase; PNMT, phenylethanolamine N-methyltransferase; ADRA1A, ADRA1B, ADRA1D, adrenergic alpha 1 receptors; GNAQ, guanine nucleotide binding protein, Q; GNA11, guanine nucleotide binding protein, alpha 11; PLCB3, phospholipase C, beta 3; ITPR1, inositol 1,4,5-triphosphate receptor, type 1; POMC, proopiomelanocortin; COMT, catechol-o-methyltransferase; MAO, monoamine oxidase; RGS 2,4,5, regulator of g-protein signaling.

Our study used a pathway-focused approach to identify common variants associated with hypertension in biologically relevant pathways, which minimizes the limitations of aforementioned alternatives to GWAS. Pathways with biological relevance to hypertension were identified first, with an emphasis on signal transduction and pathways with therapeutic targets, and the analysis was limited to only SNPs within these pathways. Subsequently, the pathways were analyzed to identify excesses of moderately significant (10^−2^>p>10^−5^) SNPs; the analysis was niether intended nor powered to detect individual SNPs reaching genome-wide significance. The pathway-focused approach has previously been used with success in three inflammatory diseases. [9] *A priori* identification of biologically-relevant pathways ensured ease of interpretation and minimized multiple-testing penalties. We applied the pathway-focused approach to study hypertension, diastolic blood pressure (DBP), and systolic blood pressure (SBP) in a sample of 1614 adult Nigerians, and identified an association for the adrenergic alpha 1 pathway.

## Methods

### Participant recruitment

Study participants were recruited from Yoruba-speaking communities in southwest Nigeria as part of a long-term study on the environmental and genetic factors underlying hypertension [10]. The sample is comprised of unrelated adults with normal or elevated blood pressure from Igbo-Ora, a rural town with a population of about 50,000; and from Idikan, a low-income neighborhood in the near-by city of Ibadan. The participants from Idikan were controls in the Africa-America Diabetes Mellitus (AADM) Study recruited from Ibadan [11]. Both towns are in similar neighborhoods.

### Ethics Statement

Both projects were reviewed and approved by the Institutional Review Board of the sponsoring US institutions (Loyola University Chicago and Howard University) and the Joint Ethical Committee of the University of Ibadan/University College Hospital, Ibadan, Nigeria. All participants signed informed consent administered in either English or Yoruba, which is the native language.

**Table 1:** Descriptive characteristics of phenotypes among study participants.

	Females	Males	All
No. (%)	940 (58.2)	674 (41.8)	1614
Age (years)	49.78±13.9	47.88±15.4	48.99±14.5
Weight (kg)	64.09±15.3	64.92±13.1	64.44±14.4
Height (m)	1.59±0.1	1.70±0.1	1.64±0.1
Body mass index (kg/m^2^)	25.21±5.8	22.49±4.2	24.07±5.4
Systolic blood pressure (mmHg)	129.96±27.7	134.12±27.0	131.69±27.5
Diastolic blood pressure (mmHg)	80.75±16.2	81.94±16.7	81.25±16.4
Hypertensives (%)	477 (50.7)	320 (47.5)	797 (49.4)
On medication (%)	240 (50.3)	159 (49.7)	399 (50.1)

Values are expressed as mean±SD.

### Phenotype measurement

A screening exam was completed by trained and certified research staff using a standardized protocol [10], [12]. Information was obtained on medical history, age, body weight and height. Body weight was measured to the nearest 0.2 kg on calibrated electronic scales, whereas height was obtained using a stadiometer consisting of a steel tape attached to a straight wall and a wooden headboard. The headboard was positioned with the participant shoeless, feet and back against the wall, and head held in the Frankfort horizontal plane and measurement taken to the nearest 0.1 cm. Body mass index (BMI) was calculated as the ratio of weight in kilograms to the square of height in meters. An oscillometric device, previously evaluated in our field settings, was used for all BP measurements [12]. Three measurements were taken three minutes apart and the average of the final two was used in the analysis. Individuals with systolic blood pressure (SBP) ≥140 mmHg, diastolic blood pressure (DBP) ≥90 mmHg or on anti-hypertensive medication at time of exam were defined as hypertensives. Participants with hypertension were offered treatment after detection at the screening exam.

**Table 2:** Pathway Association Results.

		Hypertension		DBP		SBP	
Pathway	SNPs	N Sig	P_path_	N Sig	P_path_	N Sig	P_path_
ADRA1	618	40	0.0009*	34	0.0007*	37	0.06
ADRA1, – receptors†	588	27	0.03	20	0.01	24	0.52
ADRA2	668	22	0.01	27	0.06	27	0.06
ADRB2	631	22	0.03	29	0.13	28	0.21
ADRB1	610	20	0.04	26	0.09	26	0.20
ET	415	26	0.08	18	0.23	20	0.91
DRD1A	825	42	0.09	40	0.004	47	0.76
AVP1	284	19	0.10	12	0.06	14	0.89
VIP	316	11	0.12	18	0.37	13	0.29
KATP	20	1	0.14	1	0.20	1	0.31
DRD1B	430	13	0.15	23	0.22	23	0.40
ACH	380	13	0.18	23	0.35	16	0.17
AVP2	89	1	0.18	5	0.57	6	0.25
RAA1	312	21	0.20	22	0.58	16	0.41
RAA2	363	26	0.22	26	0.55	19	0.47
PGI2	296	12	0.23	17	0.31	13	0.29
AT2C	965	49	0.23	45	0.73	46	0.85
PUR2	89	4	0.24	1	0.77	4	0.84
NA	807	33	0.25	41	0.68	42	0.71
HIST	325	14	0.27	19	0.25	16	0.12
NAK	444	38	0.28	32	0.19	28	0.11
PUR1	72	2	0.40	2	0.14	1	0.71
PX	72	4	0.45	6	0.16	4	0.05
NO	591	26	0.48	29	0.51	31	0.59
ANP	21	1	0.50	0	1.00	1	0.38
AT2A	144	9	0.51	11	0.37	8	0.15
BNP	20	1	0.52	0	1.00	1	0.40
AT2B	235	12	0.64	20	0.46	13	0.24
CNP	21	0	1.00	1	0.28	0	1.00

* p-value is significant after correction for multiple testing.

† ADRA1, -receptors is the same pathway as ADRA1, with the ADRA1 receptor genes removed from the model.

Adjustment was made for sex, age, age^2^ and BMI for all models.

### Genotyping and quality assessment

Data used in the present analysis were extracted from curated quality controlled genotype data on 1614 unrelated hypertensive or normotensive adults genotyped on the Affymetrix Genome-Wide Human SNP array 6.0. Descriptions of the genotyping and quality control of the genotype data have been provided elsewhere [13]. Briefly, 1253 DNA samples were genotyped on the Affymetrix Genome-Wide Human SNP array 6.0 at the Broad Institute, Cambridge, MA. The chip analysis provided data on 909,622 SNPs. Four samples were excluded because of discordance between reported gender and observed gender from the genotype data. Samples with inbreeding coefficients outside of four standard deviations of the mean coefficient were removed (n = 11) as well as samples sharing high IBD proportion either due to sample contamination (n = 14), duplicates (n = 18), or cryptic relatedness (n = 1). Additional samples identified as outliers based on multidimensional scaling analysis of the genome-wide IBD pairwise distances (n = 12), or clustering of missing genotypes (n = 5) were dropped. As part of quality control procedure, SNPs with a proportion of missing genotypes >0.05 (n = 34137) or with MAF <1% (n = 64955) or failing HWE test at *P*<1.0×10^−6^ (n = 16616) were all excluded. In addition, genotypes for 1,035 SNPs found to exhibit substantial deviations associated with assay plates or batch effects were dropped. The resulting quality controlled clean dataset consisted of 1,188 subjects with genotypes on 759,215 SNPs. We did not observe significant evidence of population stratification among the samples.

**Table 3:** Genes in the ADRA1 Pathway.

		Hypertension			DBP			SBP		
Gene	SNPs	N Sig	P_gene_	OR	N Sig	P_gene_	Beta	N Sig	P_gene_	Beta
*TH*	4	0	1.00	NA	0	1.00	NA	0	1.00	NA
*DDC*	84	0	1.00	NA	0	1.00	NA	2	0.15	−2.81
*DBH*	30	1	0.12	1.42	0	1.00	NA	0	1.00	NA
*PNMT*	9	3	0.004	0.77	3	0.004	−2.10	3	0.009	−3.21
*ADRA1A*	59	2	0.30	1.39	4	0.54	1.84	3	0.18	3.16
*ADRA1B*	30	8	0.005	0.57	7	0.02	−4.17	7	0.02	−4.24
*ADRA1D*	33	3	0.19	1.35	2	0.03	2.76	3	0.28	3.47
*GNAQ*	53	4	0.18	0.77	0	1.00	NA	3	0.62	3.74
*GNA11*	8	0	1.00	NA	0	1.00	NA	0	1.00	NA
*PLCB3*	3	0	1.00	NA	0	1.00	NA	0	1.00	NA
*ITPR1*	208	15	0.10	0.64	12	0.08	−4.14	11	0.77	−4.50
*POMC*	13	0	1.00	NA	1	0.33	−1.38	1	0.32	−2.27
*COMT*	25	0	1.00	NA	0	1.00	NA	0	1.00	NA
*MAOA*	28	0	1.00	NA	2	0.27	1.24	0	1.00	NA
*MAOB*	45	0	1.00	NA	2	0.12	2.74	0	1.00	NA
*RGS2*	16	2	0.21	1.23	0	1.00	NA	3	0.16	3.12
*RGS4*	19	1	0.12	2.17	0	1.00	NA	1	0.22	8.35
*RGS5*	53	1	0.61	0.85	1	0.36	−1.55	0	1.00	NA

**A**djustment was made for sex, age, age^2^ and BMI for all models.

To increase the sample size, an additional batch of 449 samples, selected from the same cohort as the first batch, was genotyped on same Affymetrix Genome-Wide Human SNP array 6.0. This batch was similarly processed using standard quality control procedure. SNPs with a proportion of missing genotypes >0.05 (n = 25090) or with MAF <1% or failing HWE test at *P*<1.0×10^−6^ (n = 68205) or with significant plate effect (n = 188) or significant differential missingness by phenotype status (n = 86) were excluded. Also, samples with inbreeding coefficients outside of four standard deviations of the mean coefficient (n = 2) and samples identified as outliers based on multidimensional scaling analysis of the genome-wide IBD pairwise distances (n = 6) were dropped. The number of samples that passed quality control was 441, of which 15 samples were replicates from the first batch. We assessed concordance between both batches of genotyping using the data on 15 samples replicated in both and observed concordance rate of 0.997876. For the present study, we combined data on the 426 unique samples from the second batch with data on 1188 samples from the first batch. Prior to merging both datasets, all excluded SNPs in batch one data were also excluded from batch two data. The final cleaned dataset thus consisted of 1614 unrelated adult hypertensive (n = 790) and normotensive (n = 824) subjects, 940 females and 674 males with 759,215 SNPs across both the 22 autosomal and X chromosomes. There was no significant evidence of population stratification.

**Table 4:** Varying R^2^ and P_SNP_ cutoffs for the ADRA1 Pathway.

Outcome	Conditions	R^2^ (LD)	P_SNP_ cutoff	P_path_
Hypertension	Default	0.5	.05	0.0009
Hypertension	Strict	0.1	.01	0.10
Hypertension	Loose	0.8	.05	0.003
SBP	Default	0.5	.05	0.06
SBP	Strict	0.1	.01	0.19
SBP	Loose	0.8	.05	0.07
DBP	Default	0.5	.05	0.0007
DBP	Strict	0.1	.01	0.03
DBP	Loose	0.8	.05	0.003

### Pathway Selection and Construction

Genes in pathways related to the renin-angiotensin-aldosterone system and vascular smooth muscle cell (VSMC) contraction were selected using KEGG (http://www.genome.jp/kegg/pathway.html) and Biocarta (http://biocarta.com/genes/allpathways.asp). Other pathways related to hypertension were selected from the literature, with an emphasis on signal transduction and pathways with therapeutic targets. A total of 28 pathways, 101 unique genes, and 4,349 unique SNPs was included in the study (Table S1). Release of calcium from the sarcoplasmic reticulum (SR) was chosen as the end-point for each pathway involved in VSMC contraction, because multiple pathways converged onto that event. Inclusion of genes (e.g. myosin light-chain, myosin light-chain kinase, etc.) involved downstream of SR calcium release did not significantly alter the results (data not shown). Literature searches were used to determine the relevant tissue-specific isoform for genes that display tissue-dependent isoform expression. Gene borders were defined using the hg-18 list from UCSC published on Plink's website (http://pngu.mgh.harvard.edu/~purcell/plink/dist/glist-hg18), and adding 20 kb to each side of the range for inclusion of regulatory regions.

**Figure 2:**
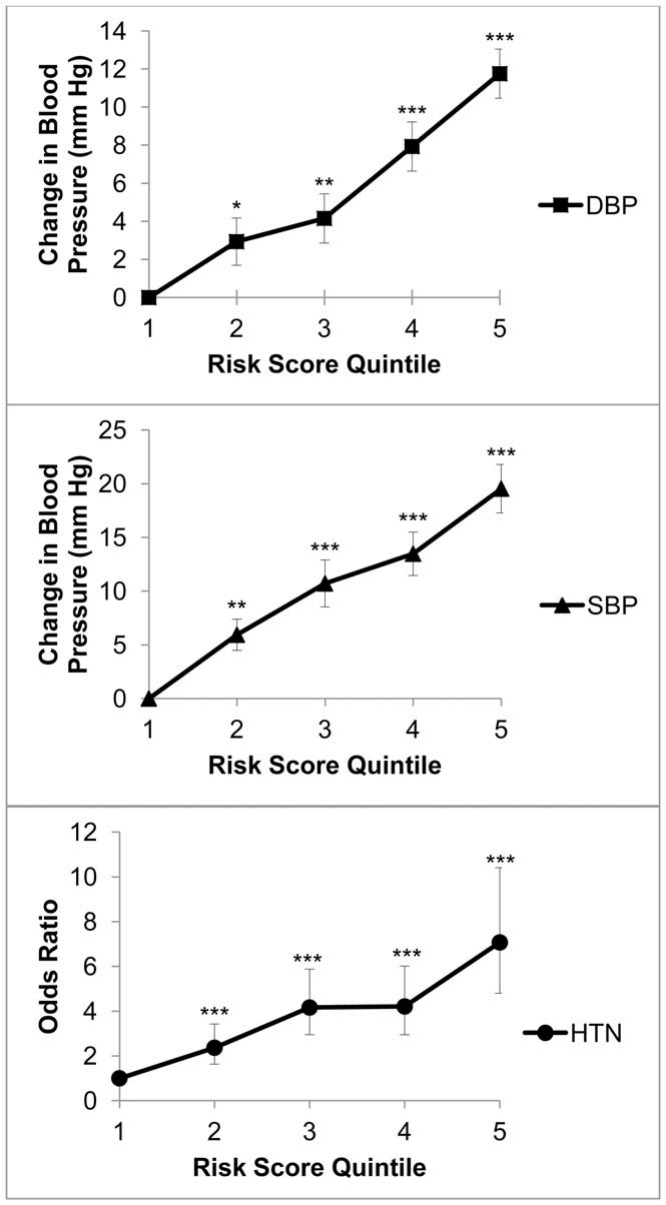
Line graphs representing the ADRA1 pathway genetic risk model for DBP, SBP, and hypertension (HTN). The risk score was divided into quintiles, with the reference category being the lowest risk score quintile. * P<0.05. ** P<0.005. ***P<0.0001.

### Statistical analyses

Association analyses were performed for blood pressure both as a dichotomous (hypertensives vs. normotensives) and a continuous trait. There were 790 hypertensives defined as those with systolic blood pressure ≥140 mmHg, diastolic blood pressure ≥90 mmHg or on medication at time of exam. When used as a continuous trait, the observed blood pressure on the 399 hypertensive individuals known to be on antihypertensive medication were adjusted by adding 15 mmHg to systolic and 10 mmHg to diastolic values [14]. Additive genetic models were used to fit logistic regression for the hypertension phenotype and linear regression for the continuous BP trait. Each SNP was coded as the count or dosage of the minor allele per genotype in both the logistic and linear regression models, and adjustment was made for sex, age, age^2^ and BMI. For the SNPs on the X chromosome, the coding involved assigning ‘1’ when the minor allele is the genotype or ‘0’ when the major allele is the genotype for males since males are hemizygous. The coding for females is same as for the autosomal SNPs. Inclusion of sex as covariate in the association analysis on the X chromosome is equivalent to stratification of the analysis by sex [15].

#### Pathway significance

Pathways were assessed for significance using the set-based method in Plink v1.07 [16], which performs well compared to other set-based tests [17]. Briefly, a threshold R^2^ value (for LD) and a P_SNP_ cutoff are specified. An association analysis is performed on the original dataset, and the most significant SNP is selected. All SNPs that have R^2^ values above the threshold value are eliminated to prevent the inclusion of multiple SNPs from the same locus. Then, the next most significant SNP is selected. The iterative process repeats until the next selected SNP does not meet the specified P_SNP_ cutoff. Next, 10,000 permuted datasets are created by swapping case and control labels, and the aforementioned process is repeated for each permuted dataset. The empirical p-value for the pathway (P_path_) is the number of times the permuted set-statistic exceeded the original dataset-statistic for that pathway. There were a total of 28 unique pathways, for a significance cutoff of P_path_<0.002.

#### Gene significance

Genes were assessed using the set-based method in Plink, using the gene as the unit rather than the pathway. There was a total of 101 unique genes, for a significance cutoff of P_gene_<0.0005.

#### SNP significance

**There was a total of 4,349 unique SNPs, for a significance cutoff of P_SNP_<1.15×10^−5^.

#### ADRA1 Pathway Genetic Risk Model

A risk score was created using the approach proposed by Morris et al [18]. Alleles for all significant SNPs within the ADRA1 pathway (40 for hypertension, 34 for DBP, and 37 for SBP) were summed and weighted equally to form the denominator (n_i_), which represents the number of total alleles. The numerator (r_i_) represents the number of unfavorable alleles for each individual. SNPs were coded so that the minor allele of risk SNPs (OR>1 or beta>0) and the major allele of protective SNPs (OR<1 or beta<0) were summed to equal r_i_, because the presence of these alleles would increase an individual's risk. An individual's score (r_i_/n_i_) was calculated as equal to the ratio of number of unfavorable alleles (r_i_) carried by the individual to the total number of alleles (n_i_). The risk score variable was subsequently categorized into quintiles for analysis in adjusted regression models.

## Results

The study population was 49% hypertensive, 58% female, had a 24.07±5.4 mean BMI (kg/m^2^), and were 48.99±14.5 mean years of age (Table 1). Pathways were tested for significance using hypertensive case status, SBP, and DBP as the outcome variables (Table 2). Only the adrenergic alpha 1 pathway (ADRA1) was significantly associated with at least one of the outcome variables after correcting for testing of 28 pathways. The ADRA1 pathway showed a stronger association with DBP (34/618 SNPs significant, P_path_<0.0007) and hypertension (40/618 SNPs significant, P_path_<0.0009) than SBP (37/618 SNPs significant, P_path_<0.06). The ADRA1 pathway consists of genes involved in epinephrine and norepinephrine synthesis, genes involved in VSMC signal transduction leading to intracellular calcium release, and major regulatory proteins (Figure 1). ADRA1 remained associated with hypertension (P_path_<0.03) and DBP (P_path_<0.01) even when the ADRA1 receptor genes (*ADRA1A*, *ADRA1B*, *ADRA1D*) were removed from the model, indicating multiple genes within the pathway were responsible for the observed association.

The ADRA1 pathway was further examined at the gene level (Table 3), including the odds ratio (OR) or beta value and p-value for the most significant SNP in each gene. *PNMT*, which catalyzes the synthesis of epinephrine from norepinephrine, displayed the strongest association with hypertension (P_gene_<0.004), DBP (P_gene_<0.004), and SBP (P_gene_<0.009) of any gene in the ADRA1 pathway. Of the three alpha 1 adrenergic receptor isoforms (*ADRA1A*, *ADRA1B*, *ADRA1D*), *ADRA1B* showed the strongest association with hypertension (P_gene_<0.005), DBP (P_gene_<0.02), and SBP (P_gene_<0.02). Multiple SNPs within both *PNMT* (3 SNPs) and *ADRA1B* (7–8 SNPs) were associated with hypertension, DBP, and SBP. None of the genes within the ADRA1 pathway had a significant P_gene_ value after correction for multiple testing. Likewise, none of the SNPs within the ADRA1 pathway had a significant P_SNP_ after multiple testing correction, and other pathways had SNPs with lower p-values than those in ADRA1 (e.g. rs929358, *ATP1A1* gene, NAK pathway for SBP), but ADRA1 was the only significant pathway by a factor of 10^2^.

Table 4 shows the effect of varying R^2^ and the P_SNP_-value cutoff on the association results for the ADRA1 pathway. Significance under strict values (R^2^ = 0.1, P_SNP_<0.01) indicates that the pathway has few SNPs associated with disease, but the associations are strong. Significance under loose values (R^2^ = 0.8, P_SNP_<0.05) indicates that the pathway has many SNPs weakly associated with disease. The ADRA1 pathway was most strongly associated with disease under intermediate values (R^2^ = 0.5, P_SNP_<0.05), suggesting a mixture of strongly associated SNPs and multiple weakly associated SNPs.

The OR for hypertension, increase in DBP, and increase in SBP were plotted against quintile of ADRA1 pathway risk score (Figure 2). OR for hypertension (7.07, 95%CI 4.08–10.41), change in DBP (11.76 mm Hg, SE 1.29), and change in SBP (19.54 mm Hg, SE 2.25) all displayed highly significant differences (p<0.0001) when comparing the lowest quintile risk score with highest quintile risk score. It is important to emphasize that two individuals with identical risk scores may have different risk alleles contributing to the score. Thus, the observed increases in OR for hypertension and blood pressure with increasing risk score may not be applicable to the individual level.

## Discussion

Our study highlights the potential of applying a pathway-focused approach to genomic association scans for hypertension. Twenty-eight pathways were examined for association with hypertension, SBP, and DBP. Of the pathways examined, only the ADRA1 pathway remained significantly associated with hypertension and DBP after correction for multiple testing. Multiple genes within the ADRA1 pathway were associated with hypertension and DBP, with the *PNMT* gene and the *ADRA1B* gene displaying the strongest associations. Other pathways involving the *PNMT* gene (ADRA2, ADRB1, ADRB2) did not reach pathway significance, indicating that ADRA1 pathway association is not an isolated effect of the *PNMT* gene. Furthermore, when all SNPs within the ADRA1 receptor genes (*ADRA1A*, *ADRA1B*, *ADRA1D*) were removed from the model, the ADRA1 pathway remained associated with hypertension and DBP, suggesting that the observed pathway association was driven by genetic variants in several genes.

Given the importance of blood pressure control to human survival, it is unlikely that the common genetic variants influencing hypertension have large effect sizes. For example, the largest hypertension GWAS to date identified 29 SNPs associated with blood-pressure and hypertension and found that each of the risk alleles for SBP and DBP were each associated with a change of less than 1 mm Hg [19]. The impact of any single SNP on blood pressure is small, but the accumulation of SNPs within a causal pathway could have a larger effect on risk for hypertension. The pathway approach used in this study examined only a fraction of the SNPs contained in the human genome. However, these SNPs were selected because they are within genes involved in pathways that have been previously linked to hypertension, which maximizes the likelihood that these SNPs could be associated with risk for hypertension. Not only does the pathway approach reduce the likelihood of false positive associations, it is also restricted solely to pathways linked to the phenotype as opposed to the genome-wide search approach of GWAS for genetic variation in blood pressure. The pathway-focused analysis can identify an accumulation of moderately significant (10−2>p>10−5) SNPs within a pathway, a feat that is not often possible using GWAS because of the high penalty caused by multiple testing. Blood pressure is a tightly regulated physiologic parameter under high selective pressure, which makes pathway-focused analysis well suited for studying the underlying genetic etiology due to its ability to detect cumulative effects of multiple subtle SNPs within a pathway. Our study focused on moderately significant SNPs within pathways with established relevance to hypertension, and it demonstrated the strengths, weaknesses, and potential for future application of the pathway approach to hypertension genomic association scans. It must be emphasized that this study was neither powered nor intended to identify SNPs that passed the genome-wide significance threshold, but rather to identify pathways containing an excess of moderately significant SNPs.

The observed ADRA1 pathway associations in our study have not been identified in previous GWASs, but they are not unprecedented in the hypertension genetics literature. None of the SNPs identified in large-scale hypertension GWAS have been within the loci for any of the genes in the ADRA1 pathway [19]–[21]. However, the *ADRA1B* receptor has been linked to systolic blood pressure in a study of 427 young Caucasians [22], and *ADRA1B* antagonists (L-765,314, prazosin, terazosin) are commonly used to treat and investigate hypertension. A locus on rat chromosome 10 mapping to the human *PNMT* locus on chromosome 17q21–q22 has been associated with blood pressure regulation in the stroke-prone spontaneously hypertensive rat [23], [24].

One possibility for this discrepancy between large-scale GWAS and our study is the differing methods used to analyze the genomic data. GWAS focus on the single nucleotide level, whereas the pathway approach identifies sets of genes containing multiple SNPs associated with disease. None of the SNPs identified in our study was significant at the genome-wide level and they would not have been detected in a GWAS, but the cumulative effect of multiple SNPs led to significance at the pathway level. The largest hypertension GWAS to date found an excess of SNPs with moderately significant (10^−2^>p>10^−5^) associations for hypertension, and these SNPs more than doubled the proportion of phenotypic variance that could be explained by significant (p<5×10^−9^) common variants [19]. The pathway approach may prove to be an effective method to identify these moderately significant SNPs. The risk score showed the impact of an excess of moderately significant risk alleles within the ADRA1 pathway (Figure 2), although the risk score used in our study may not be a good measure of risk for an individual.

Another possibility for the discrepancy between previously published reports and our study is the increased importance of ancestral genetic background when using the pathway-focused approach. The pathway approach might be population specific, because the likelihood of having the same *set* of genetic variants within a pathway is low between populations. McCarthy showed that changes in minor allele frequency drastically changes statistical power to detect a single SNP [25]. It is likely that statistical power would vary even more dramatically between populations when using the pathway approach because many SNPs are tested concurrently.

One promise of pathway analysis and genomics in general, is the ability to stratify patients based on their own genetic information rather than the generalized category of self-identified race. The controversy [26] over the response of African-Americans to angiotensin-converting enzyme (ACE) inhibitors highlights the need to further refine our understanding of the genetic determinants of hypertension and therapeutics. Variants in the angiotensinogen gene are associated with hypertension in whites [27], but not in African-Americans or Yorubas [28]. This association has stimulated further investigation into treatment response to ACE inhibitors between whites and African-Americans. The results have been equivocal [29], [30], yet ACE inhibitor usage has been avoided in some African-American patients under the presumption of racial differences in response [31]. Even if there were a true difference in response, it is likely that some African-Americans would respond better to ACE inhibitors than some European-Americans due to wide within-population variation [32]. Thus. the difficulty in predicting race specific responses to drugs demands a more finely tuned method of stratification than race. Our current study of 1,614 Yorubas is unlikely to settle any larger debates. However, we have shown that pathway focused analysis can be used to detect accumulations of moderately significant SNPs within pathways, and that the ADRA1 pathway is associated with hypertension in our sample. The use of pathway analysis to identify novel variants associated with hypertension is an initial step in refining the pathogenesis of hypertension to the individual level rather than using broader, less accurate racial categorizations.

There are several limitations to the pathway approach taken in this study. First, there is an increased need for adequate SNP coverage due to testing of a limited area of the genome. However, the plummeting cost of genomic sequencing in the near future should gradually reduce concerns over inadequate SNP coverage. Second, the nature of pathway selection is subjective on several levels: (1) the level of evidence needed for inclusion of pathways in the study, (2) the set of genes included in the study, and (3) the length of putative regulatory regions included upstream and downstream from each gene. As more hypertension pathway studies are undertaken, the set of pathways, genes, and gene regions should become more comprehensive and then a consensus list could be produced, thereby standardizing the process of selection. Third, our study may be vulnerable to issues of external validity due to the increased importance of common ancestral genetic background in pathway study populations. Our results would need to be verified in similar and other populations to determine the extent to which pathway studies can be generalized. Finally, it is also worth noting that the significance of individual SNPs in this study should be taken with caution, since a broader approach to pathway selection would have resulted in testing more SNPs with stiffer penalties for multiple testing.

We have identified an association for hypertension and DBP in the ADRA1 pathway, and we have illustrated the potential of the pathway-focused approach to investigate genetic variation in hypertension. Unlike GWAS, targeting of selected pathways cannot be used to discover new biological mechanisms of disease. However, the pathway approach has several distinct advantages over the genome-wide approach, including ease of interpretation, minimization of multiple testing, epistasis studies in larger study populations [33], and the potential to quickly translate genetic polymorphisms associated with disease into targeted therapies. The pathway approach is an excellent option for detecting moderately significant SNPs, an intriguing source of genetic variation whose importance was implicated in the largest hypertension GWAS to date [19]. Hypertension is especially amenable to the pathway approach because there are many treatment options available for hypertensive patients. Targeted therapies that operate on pathways associated with hypertension could reduce clinical uncertainty of treating hypertension. Although the results from this study must be replicated in similar populations, this pathway-focused genomic association study shows promise as a new approach to revealing the genetic underpinnings of hypertension.

## Supporting Information

Table S1 **Pathway Description.**
(DOCX)
