# Supplementary material for: Adrenergic Alpha-1 Pathway Is Associated with Hypertension among Nigerians in a Pathway-focused Analysis
Source: PLoS One. 2012 May 16;7(5):e37145. doi: 10.1371/journal.pone.0037145 (PMC3353888; doi:10.1371/journal.pone.0037145)
Supplement: Table S1 — Pathway Description. (DOCX) [file pone.0037145.s001.docx]

Supplemental Material

**Adrenergic alpha-1 Pathway is Associated with Hypertension among Nigerians in a Pathway-focused Analysis**

Nicholas P. Reder^1*^, Bamidele Tayo^1^, Babatunde Salako^2^, Adesola Ogunniyi^2^, Adebowale Adeyemo^3^, Charles Rotimi^3^, Richard S. Cooper^1^

^1^Department of Preventive Medicine and Epidemiology, Loyola University Chicago Stritch School of Medicine, Maywood, IL

^2^Department of Medicine, University of Ibadan, Ibadan, Nigeria

^3^NIH Intramural Center for Research on Genomics and Global Health, National Human Genome Research Institute, Bethesda, MD

**Short title:** ADRA1 Pathway Associated with HTN and DBP

**Please direct correspondence to:**

Nicholas Reder, Department of Preventive Medicine and Epidemiology,

Loyola University Medical Center, 2160 S. First Ave., Maywood, IL 60153.

Phone: +1 708 327 9018

Fax: +1 708 327 9009

Email: [nireder@lumc.edu](mailto:rcooper@lumc.edu)

**Supplemental Table 1: Pathway Description**

| Pathway Abbreviation | Brief Description | Description of sources |
| --- | --- | --- |
| ADRA1 | Adrenergic alpha 1 receptor, GNAQ/GNA11 | KEGG vascular smooth muscle contraction pathway [1]. |
| ADRA2 | Adrenergic alpha 2 receptor, GNAI | Zhu et al. [2] described the relationship between sympathetic pathways and hypertension. Also see Cooper et al. [3] for evidence of linkage in Nigerians. |
| NA | Na-coupled calcium channel | A pathway involving sodium and calcium channels. The pathway includes drug target genes linked to hypertension. See Johnson et al [4]. |
| ACH | Cholinergic M2 receptor, GNAI | KEGG cholinergic synapse [5]. |
| ET | Endothelin, GNAQ/GNA11 | KEGG vascular smooth muscle contraction pathway [1]. |
| PX | Purinergic P2x receptor | See Palomin-Doza et al. [6] for a study linking P2X receptor genes to blood pressure. |
| KATP | ATP-sensitive potassium channels | Sakamoto et al. [7] showed that genes in this pathway are association with blood pressure in a Japanese sample. Ellis et al. [8] showed the same in an Australian sample. |
| NO | NOS3, GC, PKG, calcium-hydrogen pump activity | KEGG vascular smooth muscle contraction pathway [1]. |
| ADRB2 | Adrenergic beta 2 receptor, GNAS, adenylyl cyclase | See Zhu et al. [2] for a review and Krushkal et al [9] for a linkage study. |
| VIP | VIPR2 receptor, GNAS | St. Hilaire et al. [10] showed that VIP inhibited aortic smooth muscle cell proliferation, Said et al.[11] showed pulmonary arterial vasoconstriction in mice without the VIP gene. VIP receptors antagonists make the pathway a druggable target. |
| PGI2 | PGI2 receptor, GNAS | KEGG vascular smooth muscle contraction pathway [1]. |
| HIST | H2 receptor, GNAS | KEGG calcium signaling pathway [12], note that histamine receptors are one of the GPCRs in the diagram. |
| PUR1 | A1 and A2 receptors, KATP channels | Activation of A2B receptors inhibits growth of aortic smooth muscle cells [13], A1A receptor overexpression leads to enhanced tubuloglomerular feedback [14]. |
| PUR2 | P2Y, GNAQ/GNA11 | Wang et al. [15] found that the P2Y receptor was associated with hypertension in Japanese men. |
| NAK | Sodium-Potassium pump, Sodium-Calcium exchange | Meguro et al. [16] found that human aortic smooth muscle cells strongly expressed the isoforms in this pathway. |
| RAA1 | Renin-Angiotensin-Aldosterone, renal mineralcorticoid receptor, Renal Na/K pumps | KEGG: Renin-Angiotensin system [17]. |
| RAA2 | Renin-Angiotensin-Aldosterone, renal mineralcorticoid receptor, ENaC channel | KEGG: Renin-Angiotensin system [17]. |
| AT2A | ATII, AT 1-7, MAS1 receptor, SHP kinase | KEGG: Renin-Angiotensin system [17]. |
| AT2B | ATII, AT 1-7, MAS1 receptor, PI3KCa, AKT, NOS3 and NOS1 | KEGG: Renin-Angiotensin system [17]. |
| AT2C | ATII, AT1r, GNAQ/GNA11 | KEGG: Renin-Angiotensin system [17]. |
| AVP1 | AVP receptor, GNAQ/GNA11 | KEGG vascular smooth muscle contraction pathway [1]. |
| AVP2 | AVP receptor, PLD | KEGG vascular smooth muscle contraction pathway [1]. |
| ANP | NPR1, PKG | KEGG vascular smooth muscle contraction pathway [1]. |
| BNP | NPR1, PKG | KEGG vascular smooth muscle contraction pathway [1]. |
| CNP | NPR2, PKG | KEGG vascular smooth muscle contraction pathway [1]. |
| DRD1A | D1-like Dopamine receptor, PKC | See Zhu et al. [2] for a review and Krushkal et al [9] for a linkage study. |
| DRD1B | D1-like Dopamine receptor, PKA | See Zhu et al. [2] for a review and Krushkal et al [9] for a linkage study. |

**Supplementary Table 2: Genes and Gene ranges**

| Gene | Pathways | N SNPs | Chr | Range |
| --- | --- | --- | --- | --- |
| *ACE* | RAA1, RAA2, AT2A, AT2B, AT2C | 17 | 17 | 58908165 58928711 |
| *ADCY1* | HIST, PGI2, VIP, CCHB2, ACH, CCHA2 | 38 | 7 | 45580649 45729239 |
| *ADCY3* | HIST, PGI2, VIP, CCHB2, ACH, CCHA2 | 31 | 2 | 24895541 24995559 |
| *ADCY6* | HIST, PGI2, VIP, CCHB2, ACH, CCHA2 | 7 | 12 | 47446241 47469087 |
| *ADCY8* | HIST, PGI2, VIP, CCHB2, ACH, CCHA2 | 149 | 8 | 131861728 132122017 |
| *ADORA1* | PUR1 | 25 | 1 | 201363458 201403156 |
| *ADORA2A* | PUR1 | 9 | 22 | 23153529 23168325 |
| *ADORA2B* | PUR1 | 18 | 17 | 15788955 15819935 |
| *ADRA1A* | CCHA1 | 59 | 8 | 26661583 26778839 |
| *ADRA1B* | CCHA1 | 30 | 5 | 159276317 159332595 |
| *ADRA1D* | CCHA1 | 33 | 20 | 4149277 4177659 |
| *ADRA2A* | CCHA2 | 14 | 10 | 112826910 112830560 |
| *ADRA2B* | CCHA2 | 3 | 2 | 96142349 96145615 |
| *ADRA2C* | CCHA2 | 6 | 4 | 3738093 3740051 |
| *ADRB2* | CCHB2 | 21 | 5 | 148186348 148188381 |
| *AGTR1* | RAA1, RAA2, AT2C | 43 | 3 | 149898347 149943480 |
| *AGTR2* | RAA1, RAA2, AT2C | 10 | X | 115215985 115220253 |
| *AGT* | RAA1, RAA2, AT2A, AT2B, AT2C | 35 | 1 | 228904891 228916959 |
| *AKT1* | AT2B | 5 | 14 | 104306731 104333125 |
| *ATP1A1* | NAK, RAA1, DRD1A | 24 | 1 | 116717358 116748919 |
| *ATP1B1* | NAK, RAA1, DRD1A | 31 | 1 | 167342570 167368584 |
| *ATP2A2* | NO | 7 | 12 | 109203414 109273280 |
| *AVPR1A* | AVP1, AVP2 | 9 | 12 | 61826482 61832857 |
| *AVPR1B* | AVP1, AVP2 | 0 | 1 | 204390905 204398105 |
| *AVP* | AVP1, AVP2 | 3 | 20 | 3011201 3013370 |
| *CACNA1C* | NA | 251 | 12 | 2032676 2677376 |
| *CACNA2D1* | NA | 184 | 7 | 81417353 81910967 |
| *CACNA2D2* | NA | 22 | 3 | 50375234 50515896 |
| *CACNB1* | NA | 8 | 17 | 34583234 34607427 |
| *CACNB2* | NA | 213 | 10 | 18469611 18870694 |
| *CACNB3* | NA | 4 | 12 | 47498778 47508991 |
| *CACNB4* | NA | 89 | 2 | 152402386 152663790 |
| *CHAT* | ACH | 36 | 10 | 50487146 50543156 |
| *CHRM2* | ACH | 61 | 7 | 136203938 136352311 |
| *CMA1* | RAA1, RAA2, AT2A, AT2B, AT2C | 11 | 14 | 24044551 24047311 |
| *CYP11B1* | RAA1, RAA2 | 11 | 8 | 143950774 143958238 |
| *CYP11B2* | RAA1, RAA2 | 13 | 8 | 143988976 143996261 |
| *DBH* | CCHA1, CCHA2 CCHB2 | 30 | 9 | 135491305 135514287 |
| *DDC* | CCHA1, CCHA2 CCHB2 | 84 | 7 | 50493627 50600648 |
| *EDN1* | ET | 19 | 6 | 12398514 12405413 |
| *EDN2* | ET | 13 | 1 | 41717032 41722884 |
| *EDN3* | ET | 25 | 20 | 57308893 57334442 |
| *EDNRA* | ET | 50 | 4 | 148621356 148685556 |
| *EDNRB* | ET | 36 | 13 | 77367616 77447665 |
| *GNA11* | CCHA1, ET, PUR2, AT2C, AVP1, DRD1A | 8 | 19 | 3045407 3072454 |
| *GNAI1* | CCHA2, ACH | 37 | 7 | 79602075 79686661 |
| *GNAI2* | CCHA2, ACH | 3 | 3 | 50248650 50271790 |
| *GNAI3* | CCHA2, ACH | 18 | 1 | 109892708 109939975 |
| *GNAQ* | CCHA1, ET, PUR2, AT2C, AVP1, DRD1A | 53 | 9 | 79525010 79836012 |
| *GUCY1A3* | NO, ANP, BNP, CNP | 29 | 4 | 156807327 156871226 |
| *GUCY1B3* | NO, ANP, BNP, CNP | 29 | 4 | 156899664 156947506 |
| *HDC* | HIST | 20 | 15 | 48321437 48345218 |
| *HRH1* | HIST | 47 | 3 | 11153778 11279939 |
| *HRH2* | HIST | 10 | 5 | 175042317 175044162 |
| *ITPR1* | CCHA1, ET, PUR2, AT2C, AVP1, DRD1A | 208 | 3 | 4510033 4864286 |
| *KCNJ11* | PUR1, KATP | 16 | 11 | 17363371 17366782 |
| *KCNJ8* | PUR1, KATP | 4 | 12 | 21809155 21819014 |
| *MAS1* | AT2A, AT2B | 7 | 6 | 160247963 160249097 |
| *NOS1* | AT2B, | 71 | 12 | 116135361 116283965 |
| *NOS3* | NO, PUR2, AT2B | 10 | 7 | 150319079 150342609 |
| *NPPA* | ANP | 13 | 1 | 11828362 11830422 |
| *NPPB* | BNP | 12 | 1 | 1 11840107 11841579 |
| *NPPC* | CNP | 10 | 2 | 232498378 232499203 |
| *NPR1* | ANP, BNP | 8 | 1 | 151917787 151933092 |
| *NPR2* | CNP | 11 | 9 | 35782405 35799728 |
| *NR3C2* | RAA1, RAA2 | 143 | 4 | 149219364 149583093 |
| *P2RX1* | PX | 8 | 17 | 3746633 376670 |
| *P2RX4* | PX | 16 | 12 | 120132046 120156292 |
| *P2RX5* | PX | 10 | 17 | 3523270 3546332 |
| *P2RX7* | PX | 40 | 12 | 120055060 120108241 |
| *P2RY1* | PUR1 | 12 | 3 | 154035425 154038533 |
| *PIK3CA* | AT2B | 10 | 3 | 180349004 180435191 |
| *PLCB3* | CCHA1, ET, PUR2, AT2C, AVP1, DRD1A | 3 | 11 | 63775697 63791604 |
| *PLD1* | AVP2 | 77 | 3 | 172801311 173010967 |
| *PNMT* | CCHA1, CCHA2 CCHB2 | 9 | 17 | 35078032 35080254 |
| *PRCP* | AT2A, AT2B | 39 | 11 | 82213056 82289205 |
| *PRKAR1A* | CCHA2, CCHB2, DRD1B | 17 | 17 | 64019704 64040505 |
| *PRKAR1B* | CCHA2, CCHB2, DRD1B | 3 | 7 | 555912 718659 |
| *PRKAR2A* | CCHA2, CCHB2, DRD1B | 3 | 3 | 48763096 48860274 |
| *PRKAR2B* | CCHA2, CCHB2, DRD1B | 23 | 7 | 106472413 106589492 |
| *PRKG1* | NO, ANP, BNP, CNP | 522 | 10 | 52420950 53725280 |
| *PTGIR* | PGI2 | 11 | 19 | 51815564 51820194 |
| *PTGIS* | PGI2 | 37 | 20 | 47553817 47618114 |
| *PTPN6* | AT2A | 5 | 12 | 6926000 6940740 |
| *RENBP* | RAA1, RAA2, AT2A, AT2B, AT2C | 6 | X | 152853916 152863426 |
| *REN* | RAA1, RAA2, AT2A, AT2B, AT2C | 24 | 1 | 202390570 202402088 |
| *SCN9A* | NA, NAK | 36 | 2 | 166759942 166876560 |
| *SCNN1A* | RAA2 | 12 | 12 | 6326273 6354976 |
| *SCNN1B* | RAA2 | 29 | 16 | 23221091 23300121 |
| *SCNN1G* | RAA2 | 10 | 16 | 23101540 23135701 |
| *SLC8A1* | NAK | 233 | 2 | 40192789 40593079 |
| *SLC8A2* | NAK | 10 | 19 | 52623734 52666934 |
| *SLC8A3* | NAK | 110 | 14 | 69580686 69725540 |
| *TH* | CCHA1, CCHA2 CCHB2 | 4 | 11 | 2141734 2149611 |
| *VIPR2* | VIP | 46 | 7 | 158513626 158630410 |
| *VIP* | VIP | 22 | 6 | 153113625 153122593 |
| *DRD1* | DRD1A, DRD1B | 24 | 5 | 174800280 174803769 |
| *PRKCA* | DRD1A | 214 | 17 | 61729387 62237324 |
| *PRKCB1* | DRD1A | 150 | 16 | 23754822 24139063 |
| *PRKCG* | DRD1A | 9 | 19 | 59077278 59102713 |
| *SLC9A3* | DRD1B | 11 | 5 | 526333 577549 |

References

1. Vascular smooth muscle cell contraction. retrieved from <http://www.genome.jp/kegg-bin/show_pathway?hsa04270>. 2011.

2. Zhu H, Poole J, Lu Y, Harshfield GA, Treiber FA, et al. (2005) Sympathetic nervous system, genes and human essential hypertension. Curr Neurovasc Res 2(4): 303-317.

3. Cooper RS, Luke A, Zhu X, Kan D, Adeyemo A, et al. (2002) Genome scan among nigerians linking blood pressure to chromosomes 2, 3, and 19. Hypertension 40(5): 629-633.

4. Johnson AD, Newton-Cheh C, Chasman DI, Ehret GB, Johnson T, et al. (2011) Association of hypertension drug target genes with blood pressure and hypertension in 86,588 individuals. Hypertension 57(5): 903-910.

5. Cholinergic synapse. retrieved from http://www.genome.jp/kegg-bin/show_pathway?hsa04725. 2011 2011.

6. Palomino-Doza J, Rahman TJ, Avery PJ, Mayosi BM, Farrall M, et al. (2008) Ambulatory blood pressure is associated with polymorphic variation in P2X receptor genes. Hypertension 52(5): 980-985.

7. Sakamoto Y, Inoue H, Keshavarz P, Miyawaki K, Yamaguchi Y, et al. (2007) SNPs in the KCNJ11-ABCC8 gene locus are associated with type 2 diabetes and blood pressure levels in the japanese population. J Hum Genet 52(10): 781-793.

8. Ellis JA, Lamantia A, Chavez R, Scurrah KJ, Nichols CG, et al. (2010) Genes controlling postural changes in blood pressure: Comprehensive association analysis of ATP-sensitive potassium channel genes KCNJ8 and ABCC9. Physiol Genomics 40(3): 184-188.

9. Krushkal J, Xiong M, Ferrell R, Sing CF, Turner ST, et al. (1998) Linkage and association of adrenergic and dopamine receptor genes in the distal portion of the long arm of chromosome 5 with systolic blood pressure variation. Hum Mol Genet 7(9): 1379-1383.

10. St Hilaire RC, Kadowitz PJ, Jeter JR,Jr. (2009) Adenoviral transfer of vasoactive intestinal peptide (VIP) gene inhibits rat aortic and pulmonary artery smooth muscle cell proliferation. Peptides 30(12): 2323-2329.

11. Said SI, Hamidi SA, Dickman KG, Szema AM, Lyubsky S, et al. (2007) Moderate pulmonary arterial hypertension in male mice lacking the vasoactive intestinal peptide gene. Circulation 115(10): 1260-1268.

12. Calcium signaling pathway. retrieved from <http://www.genome.jp/dbget-bin/show_pathway?hsa04020+5023.> 2011.

13. Dubey RK, Gillespie DG, Mi Z, Jackson EK. (1998) Adenosine inhibits growth of human aortic smooth muscle cells via A2B receptors. Hypertension 31(1 Pt 2): 516-521.

14. Oppermann M, Qin Y, Lai EY, Eisner C, Li L, et al. (2009) Enhanced tubuloglomerular feedback in mice with vascular overexpression of A1 adenosine receptors. Am J Physiol Renal Physiol 297(5): F1256-64.

15. Wang Z, Nakayama T, Sato N, Izumi Y, Kasamaki Y, et al. (2010) The purinergic receptor P2Y, G-protein coupled, 2 (P2RY2) gene associated with essential hypertension in japanese men. J Hum Hypertens 24(5): 327-335.

16. Meguro K, Iida H, Takano H, Morita T, Sata M, et al. (2009) Function and role of voltage-gated sodium channel NaV1.7 expressed in aortic smooth muscle cells. Am J Physiol Heart Circ Physiol 296(1): H211-9.

17. Renin-angtiotensin system. retrieved from <http://www.genome.jp/kegg-bin/show_pathway?hsa04614>. 2011.
